# Human oral microbiome dysbiosis as a novel non-invasive biomarker in detection of colorectal cancer

**DOI:** 10.7150/thno.49515

**Published:** 2020-09-18

**Authors:** Sheng Zhang, Cheng Kong, Yongzhi Yang, Sanjun Cai, Xinxiang Li, Guoxiang Cai, Yanlei Ma

**Affiliations:** 1 Department of Colorectal Surgery, Fudan University Shanghai Cancer Center, Shanghai, China.; 2 Department of Oncology, Shanghai Medical College, Fudan University, Shanghai, China.; 3 Guangdong Provincial People's Hospital & Guangdong Academy of Medical Science, Guangdong Lung Cancer Institute, Guangzhou, China.; 4 School of Medicine, South China University of Technology, Guangzhou, China.; 5 Department of GI Surgery, Shanghai Tenth People's Hospital Affiliated to Tongji University, Shanghai, China.

**Keywords:** colorectal cancers, colorectal adenomas, oral microbiome, 16S rRNA

## Abstract

**Background:** The oral microbiome may play an important role in colorectal carcinogenesis. However, few studies have investigated the association between oral microbiome and the development of colorectal cancer (CRC). We aimed to investigate whether oral health-colorectal tumor association has an underlying microbial basis, in the quest for novel non-invasive biomarkers for CRC.

**Methods:** We collected oral swab samples from 161 patients with CRC, 34 patients with colorectal adenoma (CRA), and 58 healthy volunteers. The oral microbiota was assessed using 16S rRNA sequencing. We characterized oral microbiome, identified microbial markers, constructed and validated colorectal tumor (CRA and CRC) classifier.

**Results:** Oral microbial composition and diversity were significantly different among the three groups, and the CRA group had the highest diversity. Analysis of the functional potential of oral microbiota demonstrated that the pathway involving cell motility was overrepresented in the CRA and CRC groups relative to that in the healthy controls. Moreover, a random forest model was constructed based on oral microbial markers, which could distinguish the colorectal tumor groups from the healthy controls and achieve a powerful classification potential in the discovery and validation cohorts.

**Conclusion:** This study suggests a potential association between oral microbiome dysbiosis and colorectal cancer. Oral microbiota-based biomarkers may be helpful in predicting the risks for the development of CRA and CRC.

## Introduction

Colorectal cancer (CRC) is the third most common cancer and the second leading cause of death among malignant tumors worldwide 1, 2. It mainly originates from an adenomatous polyp then develops into advanced colorectal adenoma (CRA) with high-grade dysplasia, and finally progresses to invasive cancer 3. Many environmental factors, such as diet and lifestyle, are crucial for the gut microbial composition and function, which can affect the host gene expression, metabolic regulation, and local and systemic immune response, thereby influencing cancer development 4. The gut microbiota has been recognized to play an important role in colorectal tumorigenesis, which may promote CRC development through inflammatory pathways, microbial metabolites, or the interference in the energy balance of cancer cells 5, 6. Our previous work suggested a potential relationship between gut microbiome and metabolome in CRC 7. Adults produce >1000 mL of saliva per day on average, almost all of which enters the gastrointestinal tract 8. The lower gastrointestinal tract is inoculated every day by approximately 10^11^ bacteria from the oral cavity, which can be detected in the oral and fecal microbiota of approximately 45% tested individuals 9. As chemical converters, microbes metabolize both dietary-acquired and host-produced nutrients 10. The resulting metabolites can promote genotoxicity and tumor suppression or progression through multiple mechanisms, such as the alteration of metabolic fluxes to promote anabolic metabolism, competitive enzymatic inhibition, and modification of signaling proteins 11. Therefore, oral microbial communities may affect the gut microbial community structure 12.

The oral microbiome diversity is correlated with several human diseases including cancers 13. Fan et al. found that oral microbiota may play a role in the etiology of pancreatic cancer 14. Another study showed that the oral microbiome composition has potential implications for the early detection and prevention of esophageal cancers 15. Microbiota dysbiosis refers to altered bacterial composition 16, and the study of oral and intestinal microbiota disorders is of great importance for exploring the mechanism of colorectal carcinogenesis 17-20. However, few studies have focused on the differences in oral microbiota profiles between patients with CRA and CRC and those of healthy individuals. These three groups are consistent with the normal-adenoma-carcinoma sequence model, which reflects the evolution process of colorectal carcinogenesis 21, 22. In this study, we hypothesize that the oral health-colorectal tumor association may have an underlying microbial basis. We further explored this potential oral-gut axis in the quest for novel non-invasive biomarkers for CRC.

## Methods

### Study design and oral sample collection

**Figure 1:**
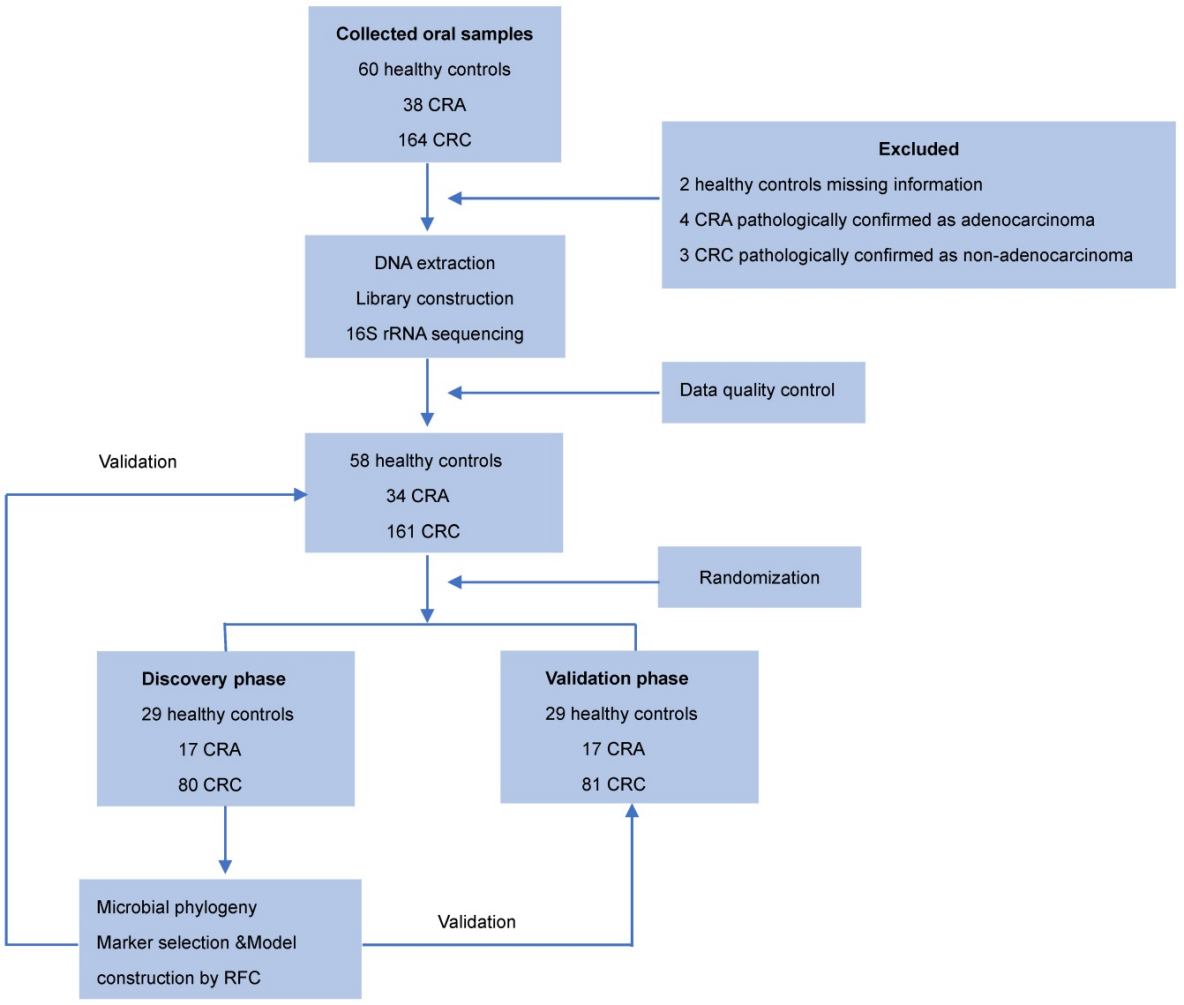
** Study design and flow diagram.** Consecutive oral samples were prospectively collected from 60 healthy controls, 38 patients with CRA, and 164 patients with CRC according to the inclusion criteria. Finally, 58 healthy controls, 34 patients with CRA, and 161 patients with CRC were included and randomly divided into discovery and validation cohorts. In the discovery phase, we characterized the oral microbiome among 80 CRC, 17 CRA, and 29 healthy controls, identified microbial markers, and constructed CRC and CRA classifiers using a random forest model. In the validation phase, 81 CRC, 17 CRA, and 29 healthy controls were used to validate the diagnostic efficacy of CRC and CRA classifiers. Furthermore, all samples (161 CRC, 34 CRA, and 58 controls) were used in another independent validation phase. CRC, colorectal cancer; CRA, colorectal adenoma.

Oral samples were prospectively collected from participants by rubbing the insides of both cheeks with a swab as described previously 23. The swab containing the specimen was placed in a sterile tube and then stored at -80 °C until further use. None of the participants had recently suffered from oral disease, and no participants were treated with any drugs according the NIH Human Microbiome Project - Core Microbiome Sampling Protocol 24. Oral samples from patients with CRC or CRA group were collected before any surgical procedure. For the CRC cohort, the exclusion criteria were: i) the diagnosis of hereditary or inflammation-associated CRC, and ii) an active preoperative treatment course. For the CRA cohort, the exclusion criteria were: i) the diagnosis of familial adenomatous polyposis, and ii) previous history of CRC. Finally, 253 eligible cases including 58 healthy controls, 34 patients with CRA, and 161 patients with CRC, were included in this study according to the recruitment process and randomly divided into the discovery phase and the validation phase **(Figure 1)**.

### Oral DNA extraction for microbiome analysis

Genomic DNA from oral swab samples was extracted using the Mag-Bind Blood & Tissue DNA HDQ 96 Kit (M6399-01, Omega, Inc, USA) according to the manufacturer's guidelines. DNA integrity and size were verified by 1.0% agarose gel electrophoresis, and DNA concentrations were determined using the NanoDrop spectrophotometer (NanoDrop, Germany).

### High-throughput 16S ribosomal RNA gene sequencing

16S ribosomal RNA (rRNA) gene amplification was performed using the primers (319F: 5′-ACTCCTACGGGAGGCAGCAG-3′; 806R: 5′-GGACTACHVGGGTWTCTAAT-3′) directionally targeting the V3 and V4 hypervariable regions of the 16S rRNA gene. To differentiate each sample and yield accurate phylogenetic and taxonomic information, the gene products were attached with forward and reverse error-correcting barcodes. The amplicons were quantified after purification. Then, the normalized equimolar concentrations of each amplicon were pooled and sequenced on the MiSeq PE300 sequencing instrument (Illumina, USA) using 2 × 300 bp chemistry according to the manufacturer's specifications.

### Sequencing data analysis

Paired-end reads were assigned to samples based on their unique barcodes and truncated by cutting off the barcode and primer sequences. Then these paired-end reads were merged using the Fast Length Adjustment of SHort reads (FLASH) tool. To obtain high-quality clean tags, the raw tags were quality filtered under specific conditions using the QIIME pipeline (Version 1.7.0) 25. Chimeric sequences were filtered using the Usearch software (Uparse v6.0.307). Sequences with a similarity threshold ≥97% were assigned to the same operational taxonomic units (OTUs) using the CD-HIT online tool (v. 4.6.1). Classification of representative sequences for each OTU was carried out and taxonomic data were then assigned to each representative sequence using the Ribosomal Database Project (RDP) classifier based on the 11.5 revision of the RDP database. We also use the NT-16S database annotation results for correction. To study the phylogenetic relationship of different OTUs, multiple sequence alignments were conducted using the PyNAST software. OTU abundance was normalized using the sample with the least sequences as a standard. To estimate the OTU richness, a rarefaction curve was designed using the Mothur software package (http://www.mothur.org/wiki/Main_Page). After the taxonomic assignment of OTUs, sequences were aligned for phylogenetic analysis. Alpha diversity was evaluated to analyze the complexity of microbial diversity for each sample through four indices (Chao1, Shannon, Simpson, and observed species) using the QIIME software. To evaluate microbial diversity between samples, beta diversity was evaluated by principal coordinate's analysis (PCoA) and cluster analysis using QIIME software. The Wilcoxon rank-sum test and Welch's *t*-test were used to compare bacterial abundance and diversity in oral swab samples from patients with CRC versus those from healthy subjects. The heatmaps were drawn based on the nonparametric Wilcox test (*p* < 0.05, *q* < 0.1) at the genus level.

### Identification of microbial OTU-based markers

A tenfold cross-validation was conducted on a random forest model to identify the optimal OTU-based markers as described previously 26. This model could specifically distinguish the CRA or CRC groups from the healthy control group.

### Statistical analysis

Associations between the clinical characteristics were performed by Pearson's Chi-square test or Fisher's exact test, as appropriate *P* values were two-tailed and adjusted using the false discovery rate (FDR). FDR values less than 0.05 were considered as statistically significant (*, <0.05; **, <0.01; ***, <0.001). All data were analyzed using GraphPad Prism v. 6.01 (Graph Pad software, lnc, San Diego, California, USA), Microsoft Excel (Microsoft Corporation, Seattle, WA, USA), and R package v. 3.3.2 (R Foundation for Statistical Computing, Vienna, Austria, http://www.R-project.org/).

## Results

### Participant information and study design

In total, 253 eligible cases including 58 healthy controls, 34 patients with CRA and 161 patients with CRC were included in this study according to the recruitment process and randomly divided into the discovery cohort and validation cohorts (**Figure 1**). There were no significant differences in the clinical characteristics, including age, gender, BMI, alcohol consumption, and smoking status among the three groups. Detailed clinical data for the studied individuals were shown in **Table 1**.

### Bacterial diversity of the oral microbiota

**Figure 2:**
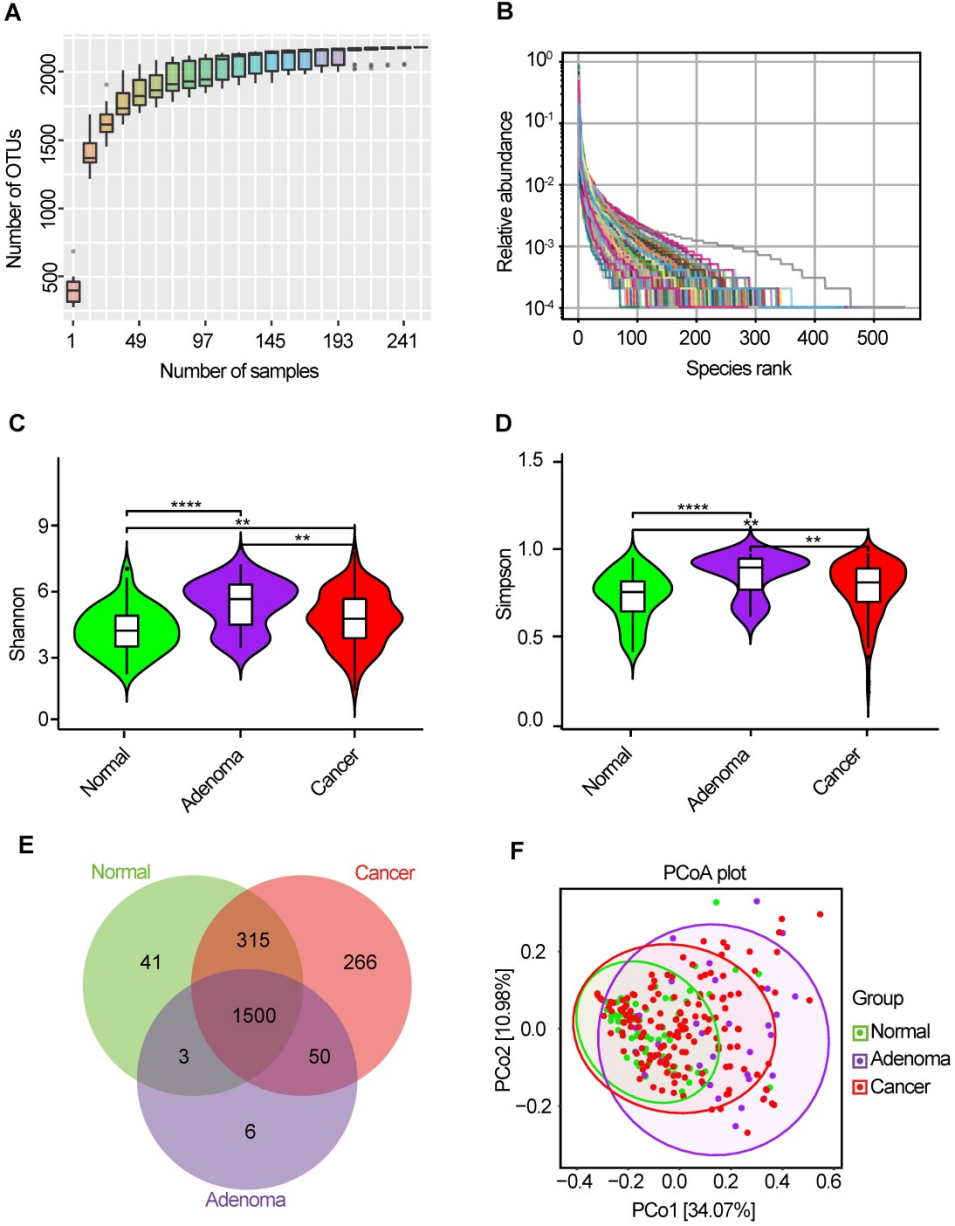
** Bacterial diversity of the oral microbiota.** (**A**) Species accumulation curve between number of samples. (**B**) The relative bacterial evenness was evaluated by the rank abundance curves. Oral microbial diversity was estimated by the Shannon index (**C**) and Simpson index (**D**). (**E**) A Venn diagram displayed the overlaps between groups. (**F**) Beta diversity was calculated using weighted UniFrac by PCoA. CRA, colorectal adenoma; CRC, Colorectal cancer; OTUs, Operational Taxonomy Units; PCoA, principal coordinates analysis.

The oral microbiota was assessed using 16S rRNA MiSeq sequencing. A total of 4,986,545 high-quality 16S rRNA gene sequences were identified, with a median read count of 17,693 (ranging from 9677 to 75385) per sample. After the taxonomic assignment, 2181 OTUs were obtained (**Table S1**). The species accumulation curve of all samples successfully reached the asymptote, supporting the adequacy of our sampling efforts (**Figure 2A**). Likewise, relative bacterial evenness was evaluated by rank abundance curves, exhibiting similar patterns in all samples (**Figure 2B**). Alpha diversity indexes were calculated to assess the differences in bacterial diversity among the three groups (**Table S2**). Bacterial diversity was analyzed using sampling-based OTUs and presented by the Shannon and Simpson indexes. The results showed that oral microbial alpha diversity was significantly higher in the CRA and CRC groups than in the healthy controls, but was lower in the CRC group than in the CRA group (**Figure 2C-D**). Moreover, the Venn diagram showed that 1500 of the total 2181 OTUs were shared among the three groups, whereas 1550 of 2140 OTUs were shared between the CRA and CRC groups. Notably, 266 OTUs were unique for the CRC group (**Figure 2E**). To display the microbiome space between samples, beta diversity was calculated using the unweighted UniFrac method and the principal coordinate nanlysis (PCoA) was performed. The results showed a gradually separated distribution of the oral microbial communities among these three groups (**Figure 2F**).

### Phylogenetic profiles of oral microbial communities

**Figure 3:**
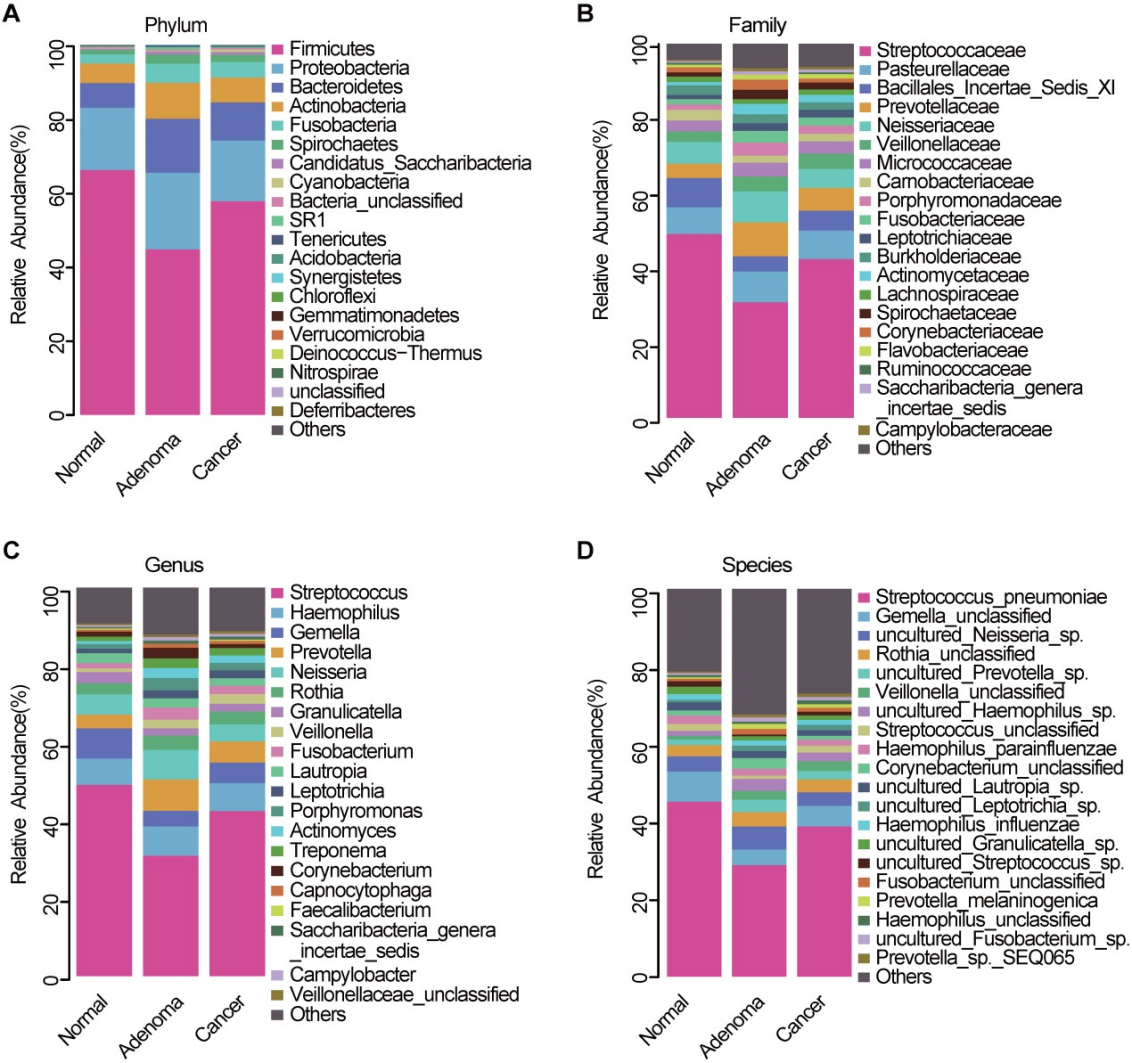
** Oral microbiota composition.** Average composition of bacterial community at the phylum (**A**), family (**B**), genus (**C**) and species (**D**) levels. CRA, colorectal adenoma; CRC, Colorectal cancer.

The average composition of bacterial communities at the phylum, family, genus, and species levels was shown in **Figure 3A-D**, respectively. Bacteroidetes, Firmicutes, Actinobacteria, Proteobacteria, and Fusobacteria were the five dominant bacterial phyla in the three groups (**Table S3**). At the phylum level, Proteobacteria, Bacteroidetes, and Fusobacteria were significantly lower in the CRC group than in the CRA group (**Figure S1A, Table S4**). At the genera level, *Fusobacterium*, *Prevotella*, and *Porphyromonas* were significantly enriched in the CRA group compared with the CRC group (**Figure S1B, Table S5**). Phylum Fusobacteria and Bacteroidetes were significantly higher in the CRA group than in the control group (**Figure S1C, Table S6**). At the genus level, *Fusobacterium*, *Prevotella*, *Porphyromonas* and *Veillonella* were significantly higher, whereas *Streptococcus*, *Gemella*, and *Megamonas* were significantly lower in the CRA group than in the control group (**Figure S1D, Table S7**). Bacterial abundance at the phylum and genus levels was also compared between the CRC and control groups. The results showed that bacterial abundance was higher in the CRC group than in the control group, especially for the phyla Fusobacteria and Bacteroidetes, and the genera *Fusobacterium, Prevotella*, and *Veillonella* (**Figure S1E-F, Table S8-9**).

### Functional and correlation network analysis of oral microbiota

**Figure 4:**
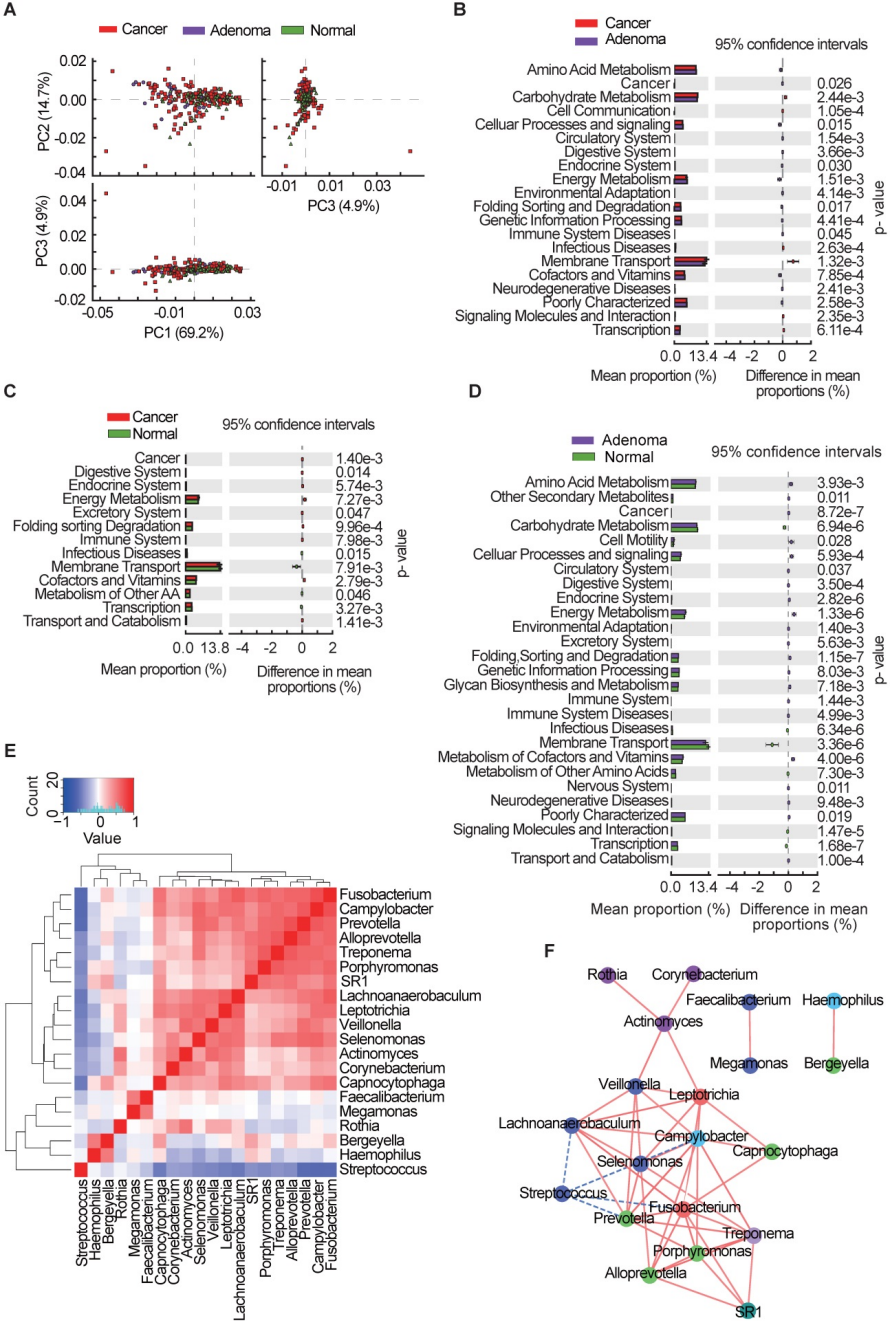
** Oral microbial functional dysbiosis in patients with CRA and CRC.** (**A**) Differential KEGG pathways were analyzed using PICRUSt, and PCoA analysis was conducted for the three groups. Significant differences between CRC and CRA group (**B**), CRC and control group (**C**), and CRA and control group (**D**) were presented respectively. (**E**) Heatmap showing Spearman correlation coefficients of 20 genera shared among CRA, CRC, and healthy controls. (**F**) Bacterial co-occurrence and anti-occurrence were investigated and presented as a network, with nodes representing bacterial genera (colored according to phylum) and edges representing interactions (red = co-occurrence, blue = mutual exclusion) at *p* < 0.01. CRA, colorectal adenoma; CRC, colorectal cancer; PCoA, principal coordinates analysis; KEGG, Kyoto encyclopedia of genes and genomes.

Microbiota imbalance induces systematic metabolic alterations 27, 28, while metabolic dysfunction can in turn influence microbiota composition 29. To study the functional and metabolic changes in oral microbial communities, all OTUs were aligned into the Phylogenetic Investigation of Communities by Reconstruction of Unobserved States (PICRUSt) built-in reference database 30, 31. A principal component analysis was then performed using the total KEGG pathways data generated from all samples (**Figure 4A**). PICRUSt analysis identified 20 KEGG pathways with significant differential abundance between the CRA and CRC groups (**Figure 4B**), 13 KEGG categories between the CRC group and the healthy controls (**Figure 4C**), and 27 KEGG categories between CRA groups and healthy controls (**Figure 4D**). As shown in **Figure 4B-D**, the membrane transport pathway was decreased in the CRA group compared with that of CRC group and control group. In addition, the pathway involved in cell motility was overrepresented while the pathway involved in carbohydrate metabolism was inhibited, in the CRA and CRC groups relative to those in the control group. The association between differential bacteria and metabolic pathways was investigated using correlation heatmaps. As the results showed, genera *Streptococcus* was negatively correlated with cell motility, endocrine system, and common oncogenic pathways, while genera *Fusobacterium, Prevotella, Leptotrichia, Selenomonas*, and* Lachnoanaerobaculum* were positively correlated with the endocrine system and oncogenic pathways (**Figure S2A-C**).

Given the significant dysbiosis of the oral microbiome in the CRA and CRC groups, as reflected by differences in bacterial composition, diversity, and function among the three groups, we focused on the 20 OTUs shared among the three groups and clustered them based on the abundance profiles (**Figure 4E**)*.* Finally, we identified two kinds of oral bacterial co-abundance groups (CAGs): the pathogen CAG (e.g. *Fusobacterium*, *Treponema* and* Porphyromonas*) and the biofilm CAG (e.g. *Streptococcus*, *Faecalibacterium*, and *Rothia*). The abundance of pathogen CAG was higher in the CRA and CRC groups than that in the control group (**Figure S3A**), while the opposite results were observed for bacterial abundance of biofilm CAG (**Figure S3B**). A stringent network analysis was also performed to obtain further insight with respect to correlative bacterial populations at the genus level. Despite the apparent dissimilarity of the microbial composition when comparing the three groups, the resulting network comprising 20 genera showed significant co-occurrence and anti-occurrence (**Figure 4F**). The oral pathogen *Fusobacterium* co-occurred with 8 other genera, namely,* Leptotrichia*, *Capnocytophaga*, *Treponema*, *SR1_genera_incertae_sedis*, *Porphyromonas*, *Alloprevotella*, *Prevotella*, and *Lachnoanaerobaculum*, many of which were categorized as oral pathogens. In contrast, *Fusobacterium* showed mutual exclusion with *Streptococcus*. We also found that the pathogenic genera belonging to the Firmicutes phylum (*Veillonella*, *Actinomyces*, *Leptotrichia*, *Selenomonas*, and* Lachnoanaerobaculum*) were closely connected to each other.

### Identification and validation of oral microbial OTU-based markers for CRA and CRC

**Figure 5:**
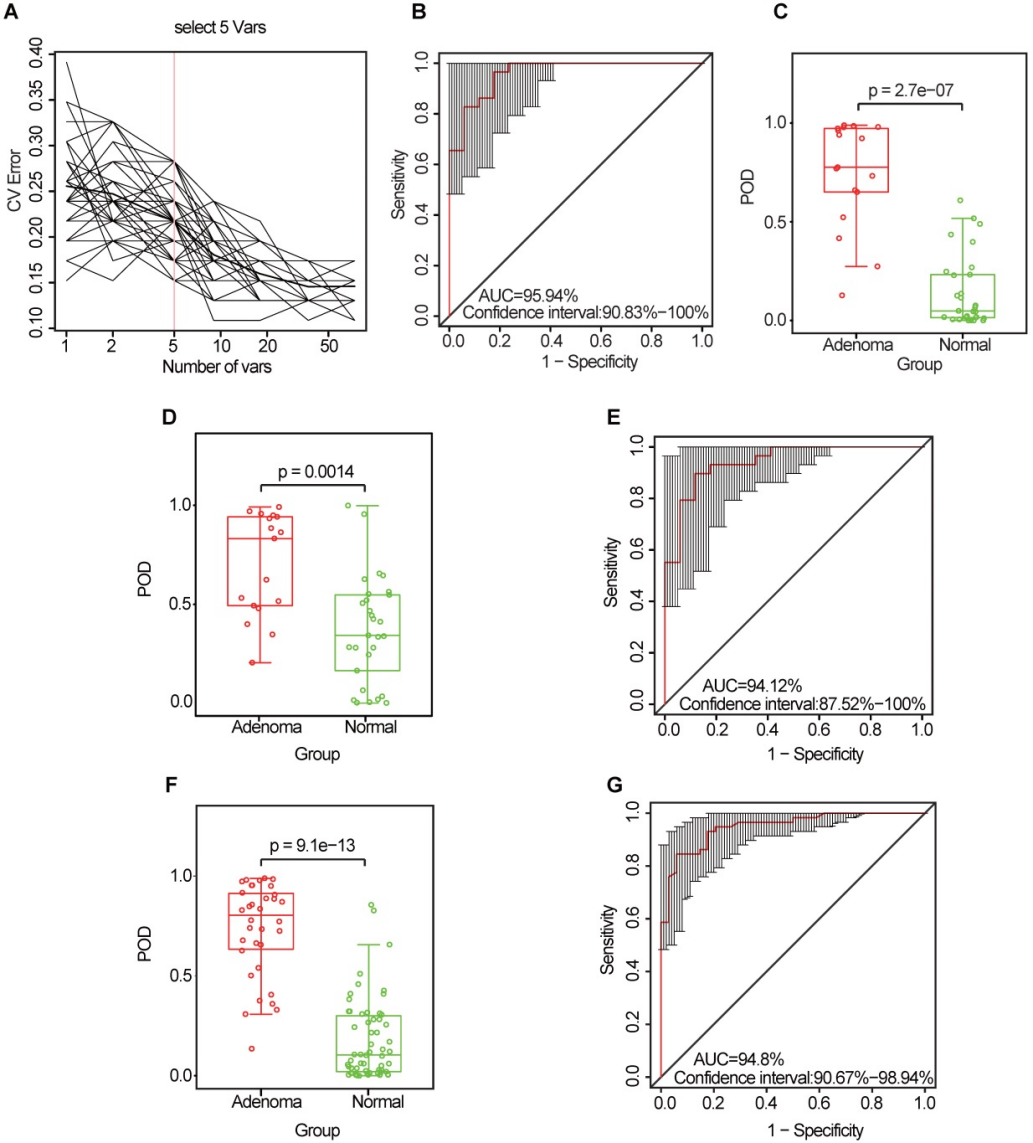
** Identification and validation of microbial OTU-based markers of CRA.** (**A**) In the discovery set, a ten-fold cross-validation was performed on a random forest model between 17 CRA samples and 29 controls. Five OTUs were selected as the optimal marker set by random forest models. (**B**) The POD index achieved an AUC value of 95.94% (95% CI: 90.83%-100%). (**C**) The POD value was significantly higher in the CRA oral samples than in the controls (*p* = 2.7 × 10^-7^). In the validation phase, the average POD value was significantly higher in the 17 patients with CRA versus the 29 controls (*p* = 0.0014) (**D**), and the POD index achieved an AUC value of 94.12% (95% CI: 87.52%-100%) (**E**). All samples including 34 CRA and 58 controls were used to verify POD reliability. The average POD value was significantly higher in CRA samples than in the controls (*p* = 9.1 × 10^-13^) (**F**), and the POD achieved an AUC value of 94.8% (95% CI: 90.67%-98.94%) (**G**). AUC, area under the curve; CV Error, the cross-validation error; CRA, colorectal adenoma; OTUs, operational taxonomic units; POD, probability of disease.

To explore the value of the oral microbiome for CRA diagnosis, a random forest classifier model was constructed to specifically distinguish the CRA from the healthy control groups: a ten-fold cross-validation was performed in the discovery phase (data from 17 CRA cases and 29 healthy controls). As a result, five OTU markers were selected as the optimal marker set (**Figure 5A, Table S10**). The probability of disease (POD) index was calculated using the identified optimal five OTU set for both the discovery cohort and the validation cohort. In the discovery phase, the POD index achieved an area under the curve (AUC) value of 95.94% (95% CI: 90.83%-100%) (**Figure 5B**), and the POD value was significantly higher in the CRA group than in the control group (*p* = 2.7 × 10^-7^, **Figure 5C**). In the validation phase (data from 17 CRA cases and 29 healthy controls), the average POD value was also significantly higher in the CRA group than in the control group (*p* = 0.0014, **Figure 5D**), and the AUC was 94.12% (95% CI: 87.52%-100%) (**Figure 5E**). Moreover, to further confirm the diagnostic potential of this random forest model, all samples (34 CRA cases and 58 healthy controls) were used to verify the POD reliability. The result also showed that the average POD value was significantly higher in the CRA group than in the control group (*p* = 9.1 × 10^-13^, **Figure 5F**), achieving an AUC value of 94.8% (95% CI: 90.67%-98.94%) (**Figure 5G**).

**Figure 6:**
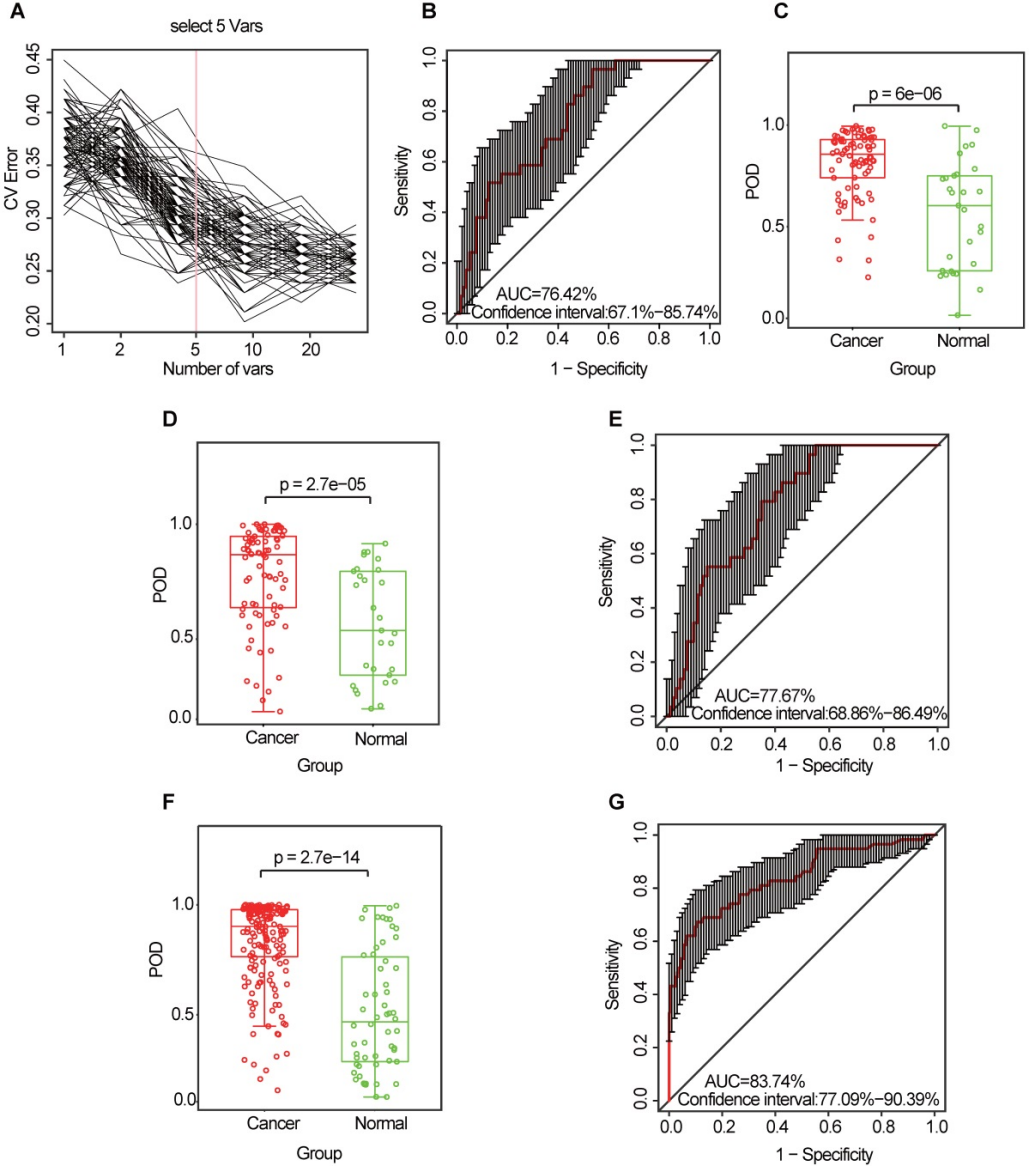
** Identification and validation of oral microbial OTU-based markers of CRC.** (**A**) A ten-fold cross-validation was conducted on a random forest model between 80 CRC samples and 29 controls in the discovery set, and five OTU markers were selected as the optimal marker set. (**B**) The POD index achieved an AUC value of 76.42% (95% CI: 67.1% to 85.74%). (**C**) The POD value was significantly higher in the CRC oral samples than in the controls (*p* = 6 × 10^-6^). In the validation phase, the average POD value was significantly higher in 81 patients with CRC versus 29 controls (*p* = 2.7 × 10^-5^) (**D**), and the POD index achieved an AUC value of 77.67% (95% CI: 68.86%-86.49%) (**E**). All samples, including 161 CRC and 58 control samples, were used to verify POD reliability. The average POD value was significantly higher in the CRC samples than in the controls (*p* = 2.7 × 10^-14^) (**F**), and the POD achieved an AUC value of 83.74% (95% CI: 77.09%-90.39%) (**G**). AUC, area under the curve; CV Error, the cross-validation error; CRC, colorectal cancer; OTUs, operational taxonomic units; POD, probability of disease.

Similarly, the diagnostic value of the CRC oral microbiome was investigated. In the discovery phase (data from 80 CRC cases and 29 healthy controls), five OTU markers were selected as the optimal marker set (**Figure 6A, Table S11**). The POD index achieved an AUC value of 76.42% (95% CI: 67.1%-85.74%) (**Figure 6B**), and the POD value was significantly higher in the CRC group than in the control group (*p* = 2.7 × 10^-5^, **Figure 6C**). In the validation phase (data from 81 CRC cases and 29 healthy controls), the average POD value was also significantly higher (*p* = 0.027, **Figure 6D**) in the CRC group than in the control group and the POD index achieved an AUC value of 63.86% (95% CI: 51.72%-75.99%) (**Figure 6E**). Furthermore, all samples (from 161 CRC cases and 58 healthy controls) were used to verify the POD reliability. This result also showed that the average POD value was significantly higher in the CRC group than in the control group (*p* = 2.7 × 10^-14^, **Figure 6F**), achieving an AUC value of 83.74% (95% CI: 77.09%-90.39%) (**Figure 6G**).

Collectively, our results indicate the powerful potential diagnostic efficacy of the POD index based on oral microbial markers from patients with CRA or CRC.

## Discussion

Accumulating evidence shows that the oral microbiome is capable of ectopic colonization and producing an extraordinarily wide range of microbial metabolites with the potential to promote tumorigenesis via modulation of pathways related to energy homeostasis, nutritional intake, and immunologic balance 32-34. Farrell et al. observed associations between variations of patients' oral microbiota with pancreatic cancer and chronic pancreatitis 35. Lu et al. identified microbiota dysbiosis of tongue coat in patients with liver carcinoma, which may provide novel and non-invasive potential diagnostic biomarker of liver carcinoma 36. Another study have shown that a high abundance of oral commensal bacteria such as *Corynebacterium* and* Kingella* correlated with a decreased risk of head and neck squamous cell cancer 37. Although there are some studies on the relationship between oral microbiota and CRC, the results are not consistent 17. In this study, we aimed to delineate the community structure and function of the oral microbiome from patients with CRA and CRC and to further establish diagnostic markers for CRA and CRC based on oral microbial OTUs.

First, we evaluated the oral microbiome diversity and found that CRC group and CRA group have a higher diversity than healthy controls. Notably, the oral microbiota of CRA group exhibited the highest diversity. It was reported that oral bacterial diversity is higher in oral diseases, such as in periodontal disease 38. By hematogenous and enteral routes, oral pathogenic bacteria can translocate to the gastrointestinal tract and induce various gastrointestinal diseases, which suggested that the higher diversity of oral microbiota in CRA group may be a high risk for gastrointestinal tumorigenesis 39. *Fusobacterium* is known to be associated with colorectal adenomas and carcinoma 40-42. In this study, we found an enrichment of* Fusobacterium* in the oral cavity of patients with colorectal tumors, especially in CRA group. Therefore, we speculate that the enrichment of Fusobacterium in the oral cavity may lead to its colonization in the colonic mucosa and further promote colorectal carcinogenesis, which indicates that *Fusobacterium* may serve as a “driver” in colorectal carcinogenesis 43. By contrary, *Fusobacterium* may serve as a “passenger” according to the classic “driver-passenger model” by Tjalsma et al. 44. Thus, the definite role of this oral species to CRC pathogenesis is still a matter of debate. Interestingly, the abundance of *Fusobacterium* was significantly lower in the CRC group than in the CRA group. Several studies have shown that* Fusobacterium* was enriched in the tumor tissue of CRC compared to CRA 43, 45, but different sample types and different detection methods may result in ambiguous results. In this study, the sample type was oral swab rather than tumor tissues. We speculated that the abundance of *Fusobacterium* in oral cavity may not be consistent with that in the tumor tissues. Moreover, *Fusobacterium* may involve in colorectal carcinogenesis in the adenoma stage, and the increase of *Fusobacterium* in oral cavity may be a specific clinical manifestation of CRA stage. This inconsistency is strange and interesting, and the mechanism involved this inconsistency need to be further studied. Furthermore, we found a significant change in the oral bacteria composition of the CRC group relative to that of the healthy controls. Flemer et al. found that several oral taxa were differentially abundant in CRC compared with controls in western populations 23. However, the differentiated composition of oral microbiome in our study is distinct from that of Flemer et al., which may be due to the different dietary structure and different races of the subjects. Together, these results indicate a significant global shift in the oral microbiota among the three groups.

Next, we conducted a functional analysis using PICRUSt. The results showed that the membrane transport pathway was decreased in the CRA groups and CRC groups compared with that in the healthy controls, which may have a potential impact on the anti-tumor immune response, such as the response mediated by bacterial outer membrane vesicles 46. In contrast, the cell motility pathway was found to be overrepresented in the CRA and CRC groups, which can promote tumor invasion and migration 47. Moreover, we identified two kinds of CAGs (pathogen CAGs and biofilm CAGs) from the top 20 bacterial genera among the three groups. Bacteria from the pathogen CAG such as *Fusobacterium*, *Treponema* and* Porphyromonas* were previously shown to be involved in the late colonization of oral biofilms and human diseases including CRC and juvenile periodontitis 48-51. Since the pathogenicity of this type of bacteria is more worthy of attention, we define them as the pathogen CAGs. In addition, several genera such as *Streptococcus, Faecalibacterium, and Rothia* existed in the early formation of tooth biofilm, which play a non-pathogenic role in healthy tooth pockets or have potential probiotic effects 52. Accordingly, we define them as biofilm CAGs. *Streptococcus*, a biofilm CAG, has promising results in treatment of halitosis, which often concurrent with digestive tract microbial disorders during the colorectal carcinogenesis 53, 54. In this study, the decrease of *Streptococcus* in the oral cavity of CRA group may contribute to microbiota dysbiosis-associated colorectal carcinogenesis. Interestingly, the stringent network analysis showed that *Fusobacterium* (pathogen CAG) and *Streptococcus* (biofilm CAG) are mutually exclusive, indicating that there may be a biological antagonism between the two CAGs. Overall, these data further suggest an oral microbiota dysbiosis in CRC and CRA groups relative to healthy controls.

Finally, we identified specific oral microbial markers to distinguish CRA or CRC patients from healthy controls and verified their diagnostic efficacy using random forest classification models. The results show that the classifier based on the five optimal OTU markers can effectively distinguish CRA or CRC patients from healthy controls in discovery cohort and validation cohort. These findings suggest that biomarkers based on the oral microbiota may help predict the risk of CRA and CRC. Hong et al. found that microbiome dysbiosis in early aberrant crypt foci legion can be used as a biomarker for potential CRC development 55. However, colonoscopy used in the study of Hong is an invasive method, whilst oral swab in our study is non-invasive that makes patients more compliant.

Nevertheless, there are some limitations about this study. First, we just compared the difference in oral microbiota compositions among the three groups, rather than do some research about the potential mechanism involved in colorectal carcinogenesis. Second, we just conducted a single-center study instead of a multi-center research, while the composition and activity of gut microbiota depends on many factors. The biomarkers are strongly ethnicity-dependent and should be validated in wide range of population. Third, metabolomics detection techniques, which can clarify the alteration of oral metabolites, may help us better understand the microecosystem network of oral microbial dysbiosis. Finally, methods of collecting oral microbes mainly include tongue coating, saliva, oral wash, and oral swabs 56. In this study, only oral swabs were used to collect samples, which may lead to biased results due to uneven distribution of oral microbiome.

## Conclusions

Oral microbial dysbiosis was found to be a common state in patients with CRA and CRC. Dysbiosis of the oral pathogen CAG (e.g. *Fusobacterium, Treponema and Porphyromonas*) and the biofilm CAG (e.g. *Streptococcus, Faecalibacterium, and Rothia*) may be important risks for colorectal carcinogenesis. Oral microbiota-based biomarkers can serve as a promising non-invasive tool for the detection of CRA and CRC. Further studies, such as constructing the special xenograft based on the target microbiota for *in vivo* and *in vitro* study, are needed to dissect the mechanisms of oral microbiota dysbiosis in colorectal carcinogenesis.

## Supplementary Material

Supplementary figures.

Supplementary tables.

## Figures and Tables

**Table 1:** Clinical characteristics of the enrolled participants

Characteristics	Healthy control (n = 58)	CRA(n = 34)	CRC (n = 161)	*P* value
Age (mean ± SD)	50.71±11.34	51.88±7.67	59.25±10.71	0.114*
BMI (mean ± SD) kg/m^2^	22.98±2.22	22.53±1.53	22.49±1.78	0.678*
**Sex**				
Male	31 (53.4%)	20 (58.8%)	107 (66.5%)	0.192^#^
Female	27 (46.6%)	14 (41.2%)	54 (33.5%)	
**Smoking status**				
Never smoker	48 (82.8%)	30 (88.2%)	120 (74.5%)	0.163^#^
Former smoker	9 (15.5%)	2 (5.9%)	36 (22.4%)	
Current smoker	1 (1.7%)	2 (5.9%)	5 (3.1%)	
**Alcohol consumption**				
Never drink	33 (56.9%)	20 (58.8%)	98 (60.9%)	0.525^#^
<1 standard drink per day	18 (31.0%)	11 (32.4%)	55 (34.1%)	
≥1 standard drink per day	7 (12.1%)	3 (8.8%)	8 (5.0%)	
**Diabetes**				
Yes	0 (0%)	0 (0%)	23 (14.3%)	
No	58 (100%)	34 (100%)	138 (85.7%)	
**Tumor location**				
Rectum	-	17 (50.0%)	78 (48.5%)	0.984^#^
Distal colon	-	10 (29.4%)	48 (29.8%)	
Proximal colon	-	7 (20.6%)	35 (21.7%)	
Tumor size (mean ± SD)	-	0.8±0.3 (cm)	4.1±1.5 (cm)	
**TNM stage**				
Stage I	-	-	24 (14.9%)	
Stage II	-	-	66 (41.0%)	
Stage III	-	-	60 (37.3%)	
Stage IV	-	-	11 (6.8%)	

*One-way analysis of variance (ANOVA); #Pearson Chi-square test.

## Abbreviations

CRC: colorectal cancer
CRA: colorectal adenoma
OTUs: operational taxonomic units
PCoA: principal coordinate analysis
PICRUSt: Phylogenetic Investigation of Communities by Reconstruction of Unobserved States
CAGs: co-abundance groups
POD: probability of disease
AUC: area under the curve
