# Supplementary material for: Human oral microbiome dysbiosis as a novel non-invasive biomarker in detection of colorectal cancer
Source: Theranostics. 2020 Sep 18;10(25):11595–606. doi: 10.7150/thno.49515 (PMC7545992; doi:10.7150/thno.49515)
Supplement: Supplementary file 1 — Supplementary figures. [file thnov10p11595s1.pdf]

Supplementary figures and table legends

Figure S1. Phylogenetic profiles of oral microbes among patients with CRC (n = 161) and CRA (n = 34) and healthy controls (n = 58). The microbial communities at the phylum (A) and genus (B) levels in CRA versus CRC are represented. The microbial communities at the phylum (C) and genus (D) levels in CRA versus controls and in CRC versus controls (E and F, respectively) are also represented. CRA, colorectal adenoma; CRC, colorectal cancer.

Figure S2. Associations of oral microbiota with inferred functions. The heatmaps show partial Spearman correlation coefficients between 32 genera and differential KEGG pathways in CRC versus controls (A), between 9 genera and differential KEGG pathways in CRA versus controls (B), and between 4 genera and differential KEGG pathways in CRC versus CRA (C). CRA, colorectal adenoma; CRC, colorectal cancer; KEGG, Kyoto encyclopedia of genes and genomes.

Figure S3. Relative abundance of two CAGs in oral samples of each group. (A). Relative abundance of pathogen CAG. (B) Relative abundance of biofilm CAG. n (control) = 58, n (CRC) = 161, n (CRA) = 34. CRA, colorectal adenoma; CRC, colorectal cancer; CAGs, co-abundance groups.

A

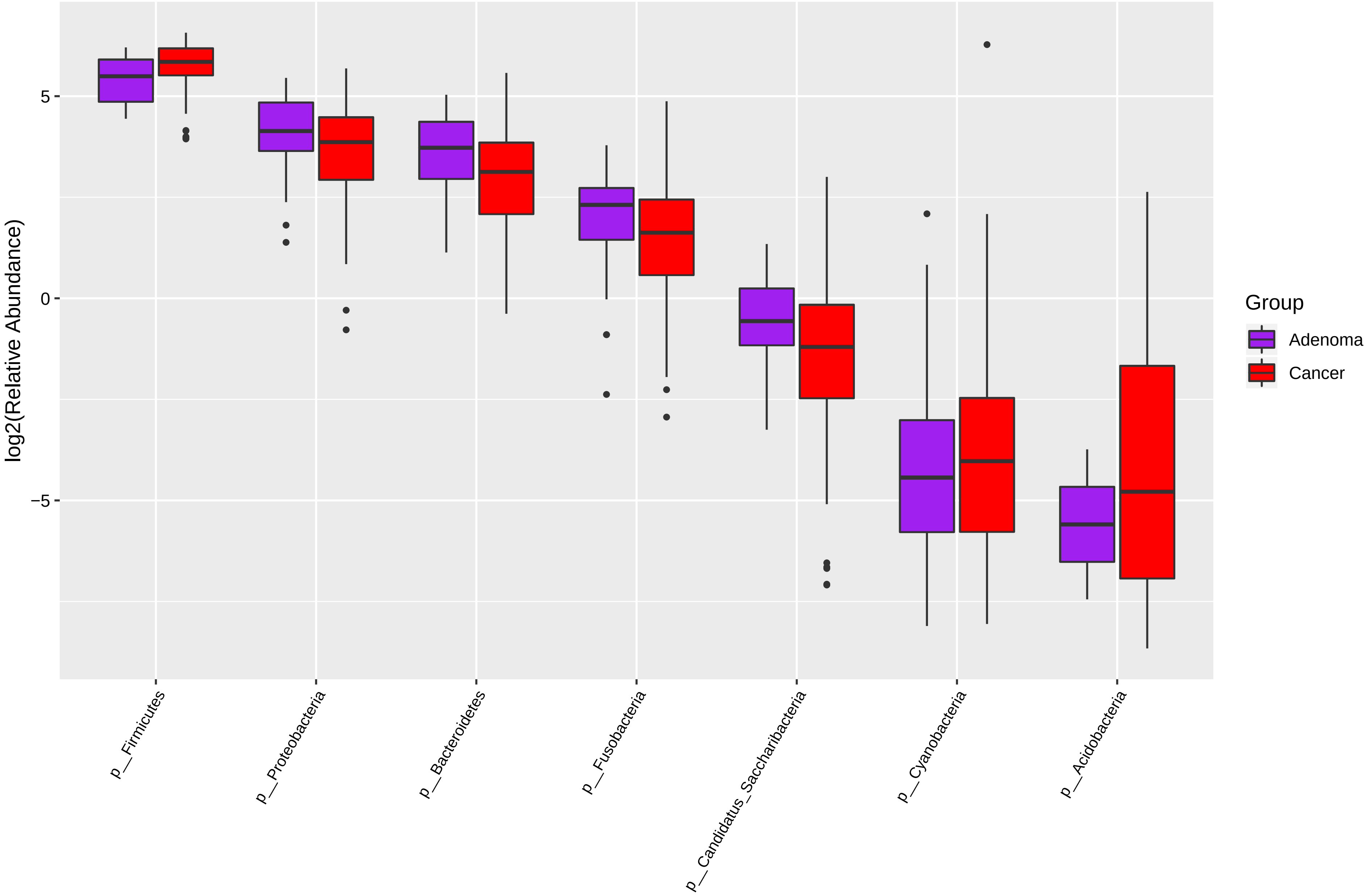
log2(Relative Abundance)
5
0
−5
p__Firmicutes
p__Proteobacteria
p__Bacteroidetes
p__Fusobacteria
p__Candidatus_Saccharibacteria
p__Cyanobacteria
p__Acidobacteria
Group
Adenoma
Cancer

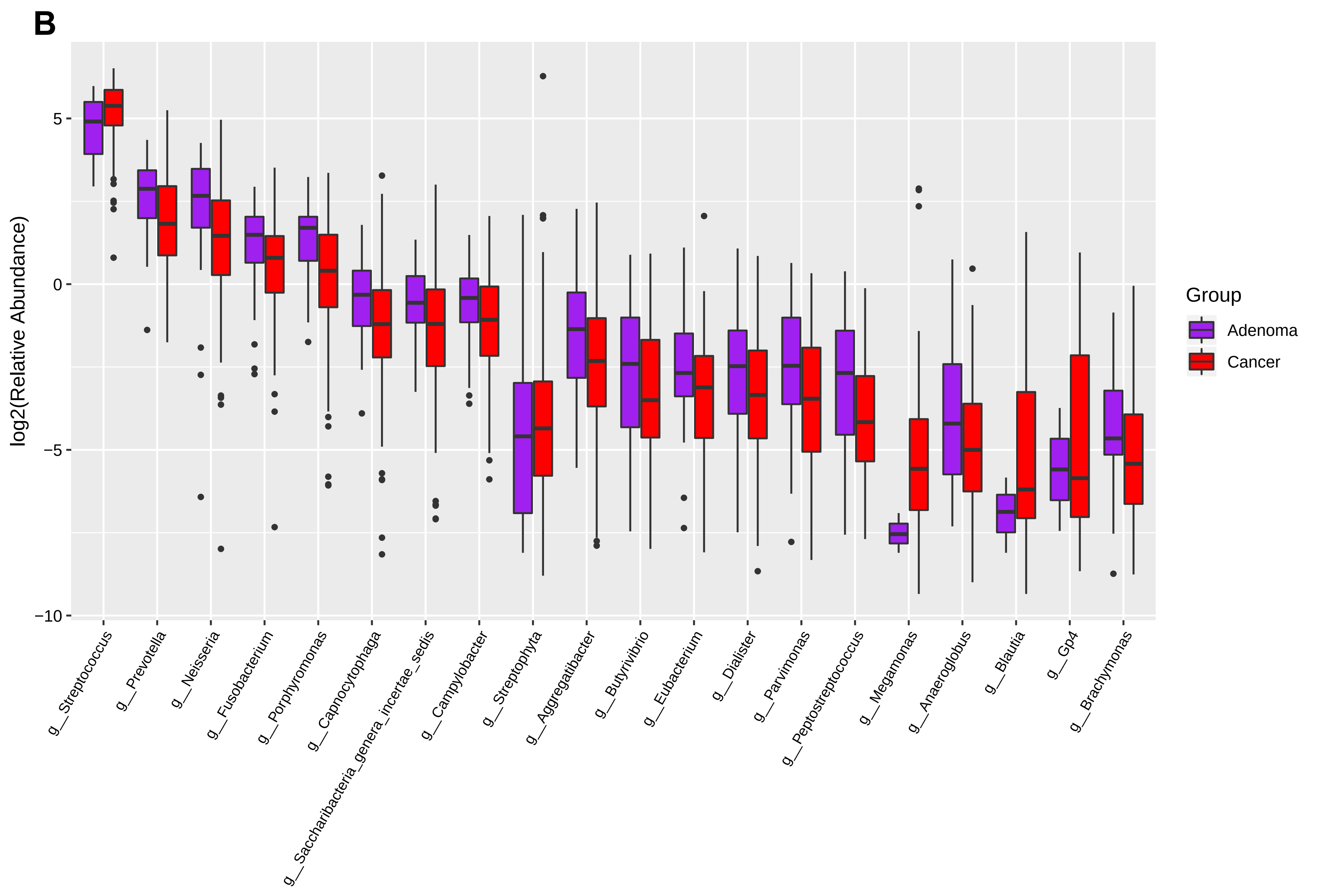
B
log2(Relative Abundance)
5
0
-5
-10
g__Streptococcus
g__Prevotella
g__Neisseria
g__Fusobacterium
g__Porphyromonas
g__Capnocytophaga
g__Saccharibacteria_genera_incertae_sedis
g__Campylobacter
g__Streptophyta
g__Aggregatibacter
g__Butyrivibrio
g__Eubacterium
g__Dialister
g__Parvimonas
g__Peptostreptococcus
g__Megamonas
g__Anaeroglobus
g__Blautia
g__Gp4
g__Brachymonas
Group
Adenoma
Cancer

C

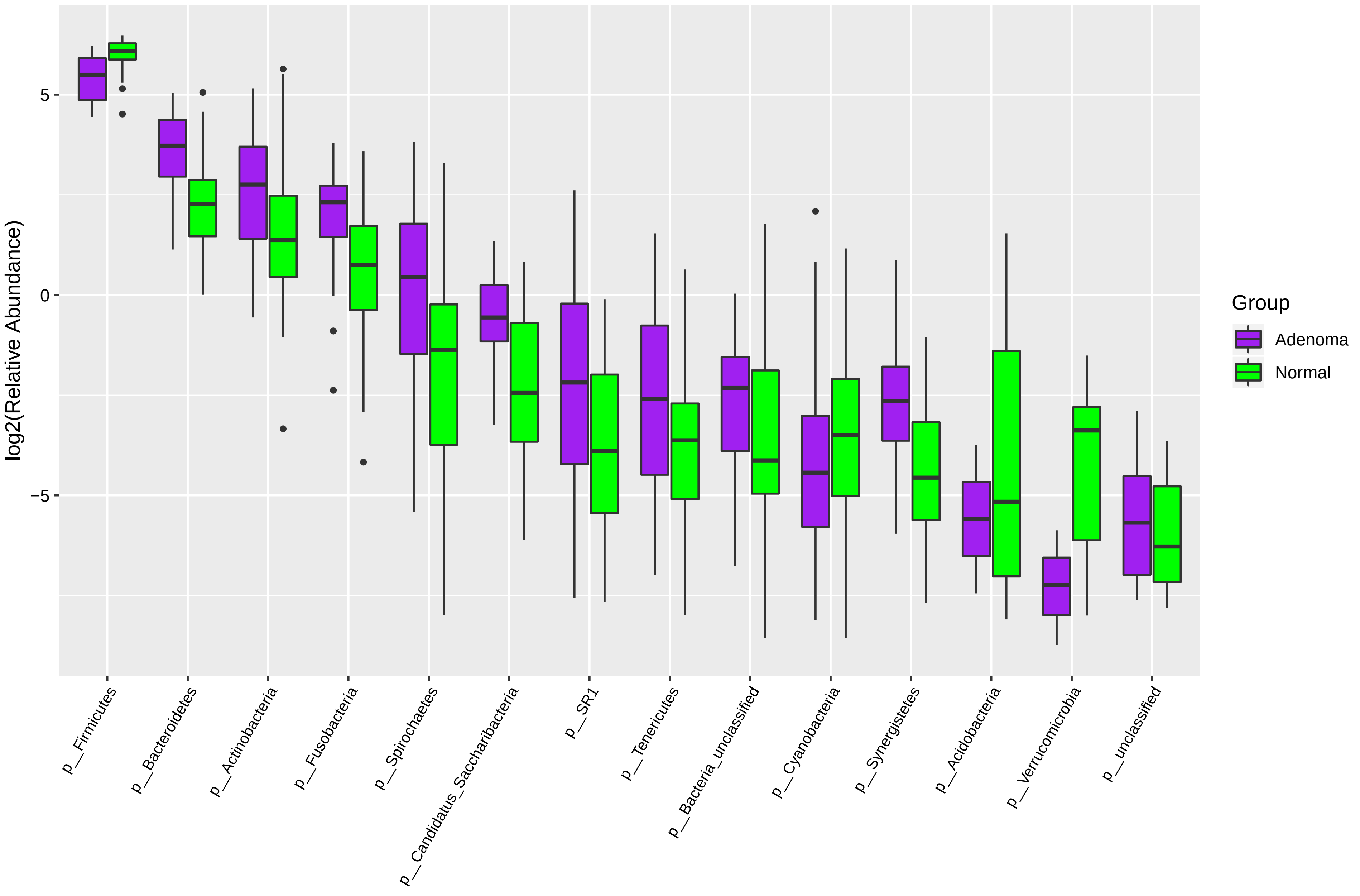
log2(Relative Abundance)
5
0
−5
p__Firmicutes
p__Bacteroidetes
p__Actinobacteria
p__Fusobacteria
p__Spirochaetes
p__Candidatus_Saccharibacteria
p__SR1
p__Tenericutes
p__Bacteria_unclassified
p__Cyanobacteria
p__Synergistetes
p__Acidobacteria
p__Verrucomicrobia
p__unclassified
Group
Adenoma
Normal

D

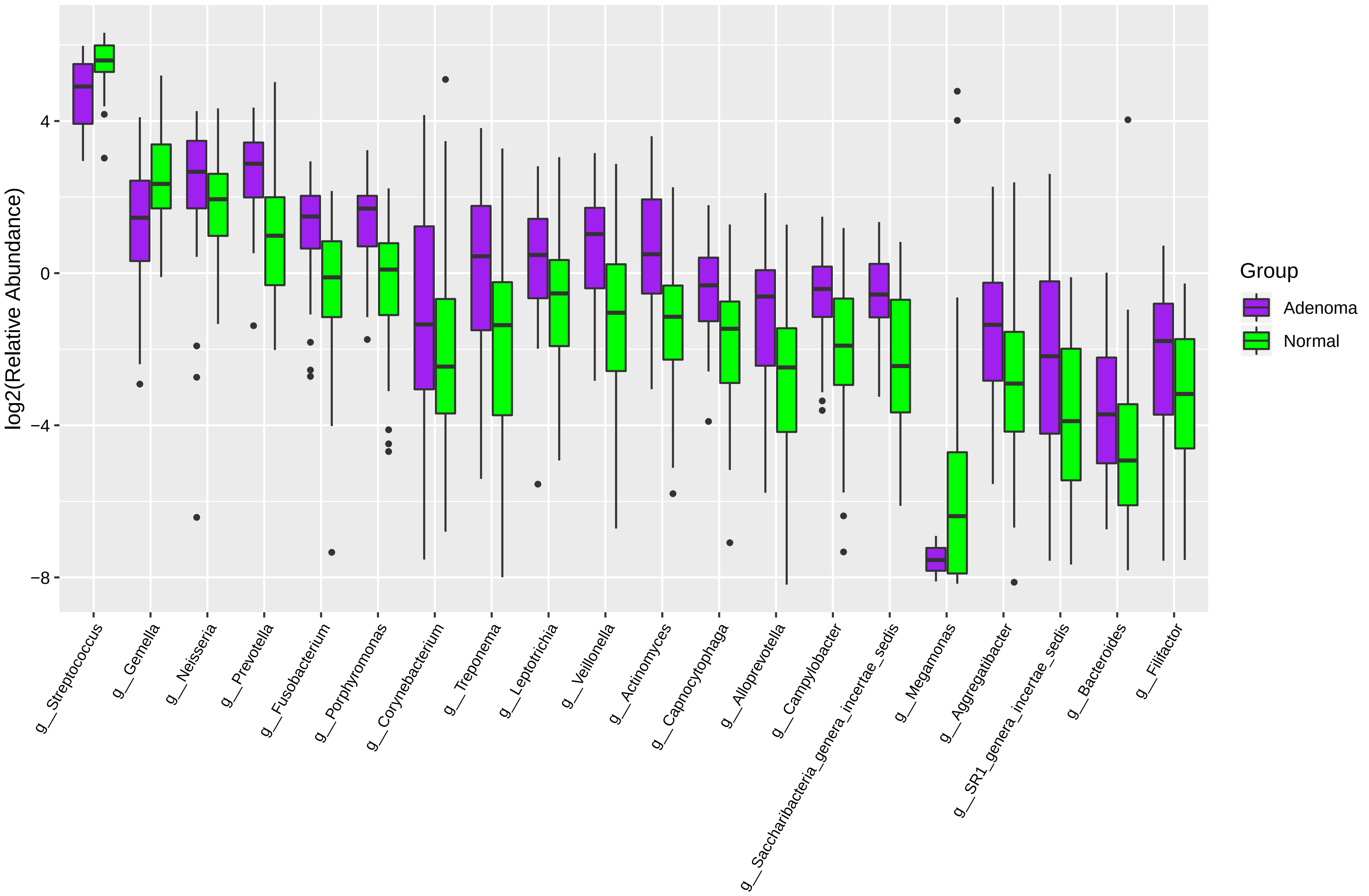

log2(Relative Abundance)
4
0
-4
-8
g__Streptococcus
g__Gemella
g__Neisseria
g__Prevotella
g__Fusobacterium
g__Porphyromonas
g__Corynebacterium
g__Treponema
g__Leptotrichia
g__Veillonella
g__Actinomyces
g__Capnocytophaga
g__Alloprevotella
g__Campylobacter
g__Saccharibacteria_genera_incertae_sedis
g__Megamonas
g__Aggregatibacter
g__SR1_genera_incertae_sedis
g__Bacteroides
g__Filifactor
Group
Adenoma
Normal

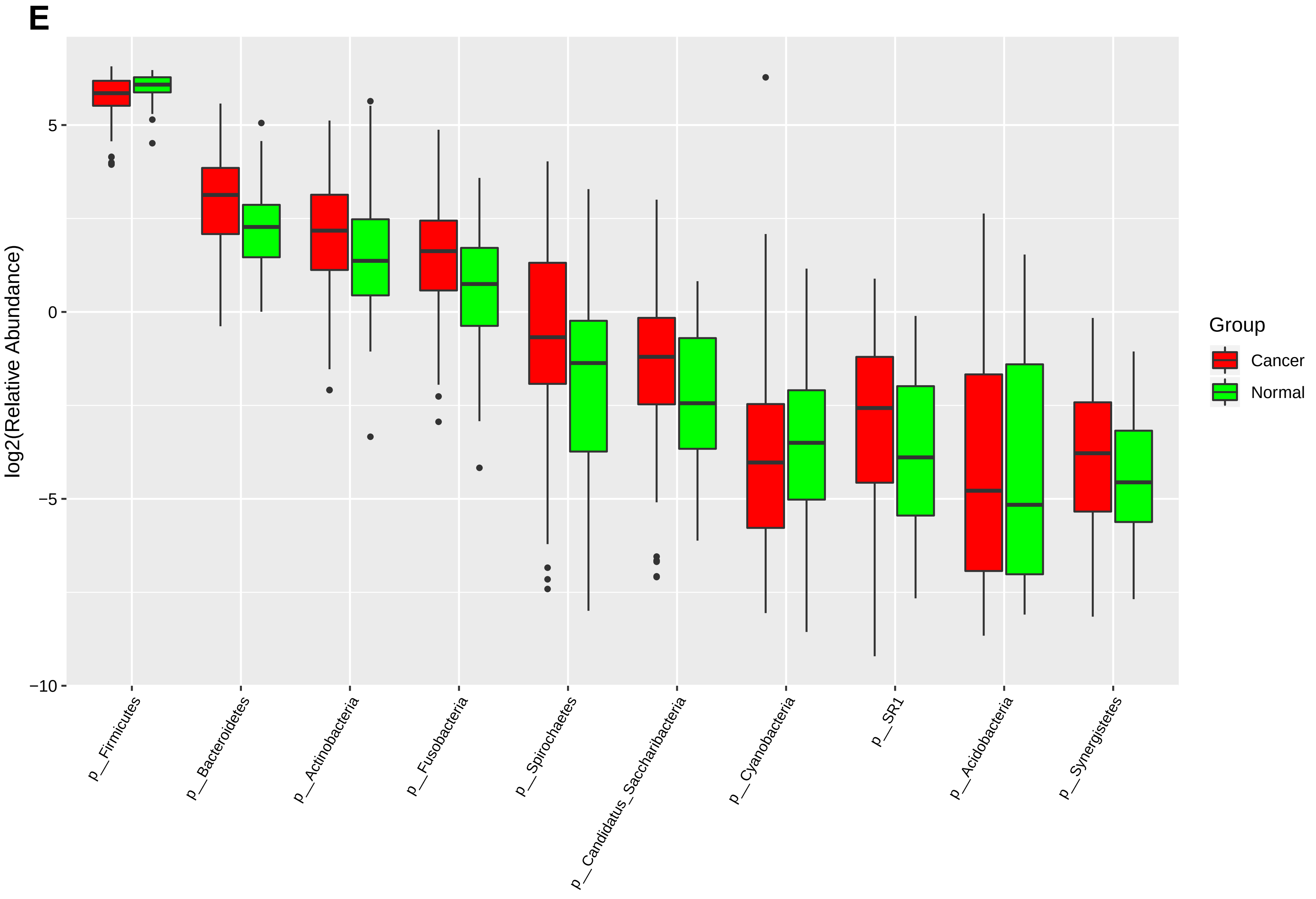
E
log2(Relative Abundance)
5
0
−5
−10
p__Firmicutes
p__Bacteroidetes
p__Actinobacteria
p__Fusobacteria
p__Spirochaetes
p__Candidatus_Saccharibacteria
p__Cyanobacteria
p__SR1
p__Acidobacteria
p__Synergistetes
Group
Cancer
Normal

F

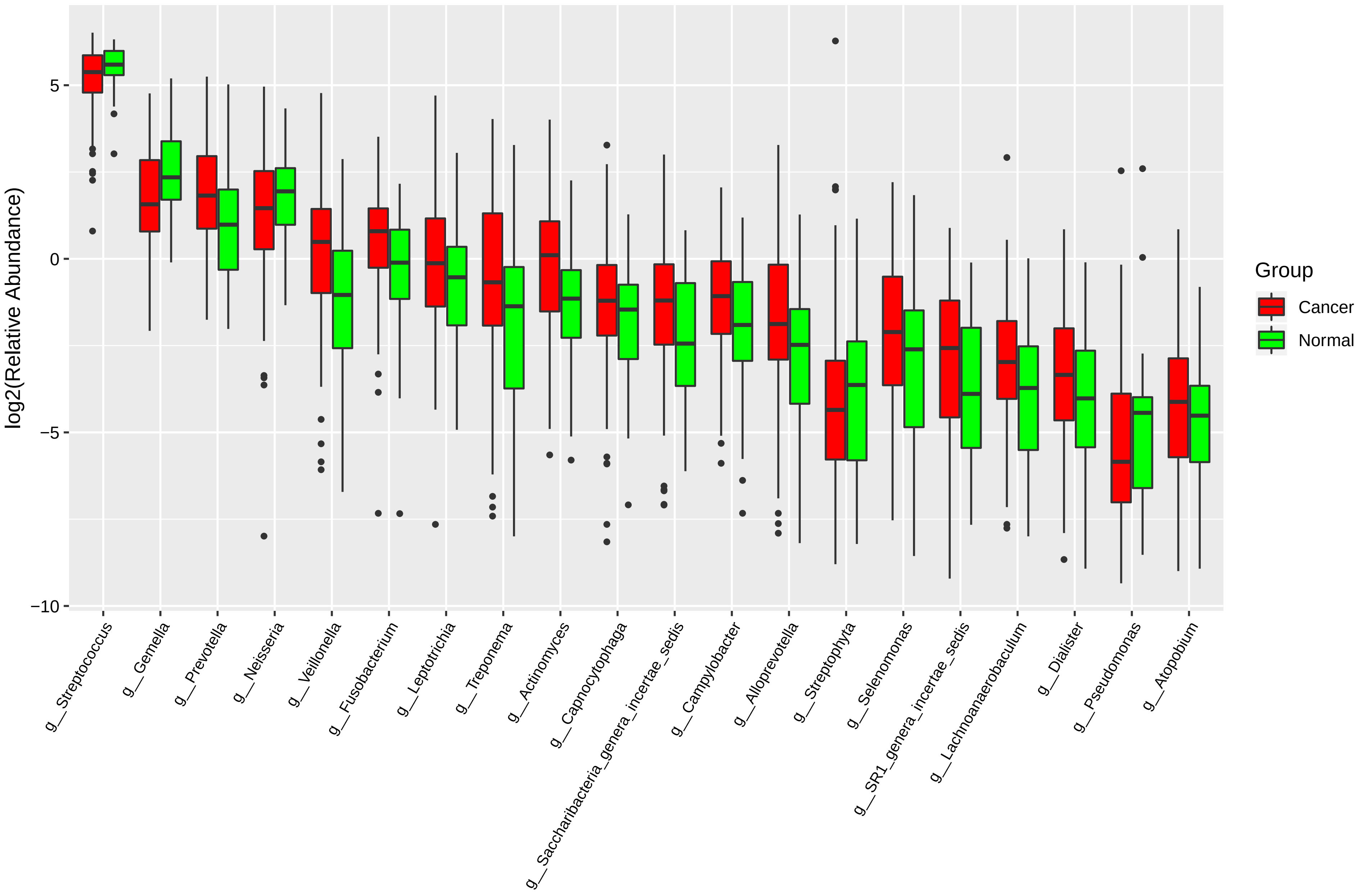

log2(Relative Abundance)
5
0
−5
−10
g__Streptococcus
g__Gemella
g__Prevotella
g__Neisseria
g__Veillonella
g__Fusobacterium
g__Leptotrichia
g__Treponema
g__Actinomyces
g__Capnocytophaga
g__Saccharibacteria_genera_incertae_sedis
g__Campylobacter
g__Alloprevotella
g__Streptophyta
g__Selenomonas
g__SR1_genera_incertae_sedis
g__Lachnoanaerobaculum
g__Dialister
g__Pseudomonas
g__Atopobium
Group
Cancer
Normal

A

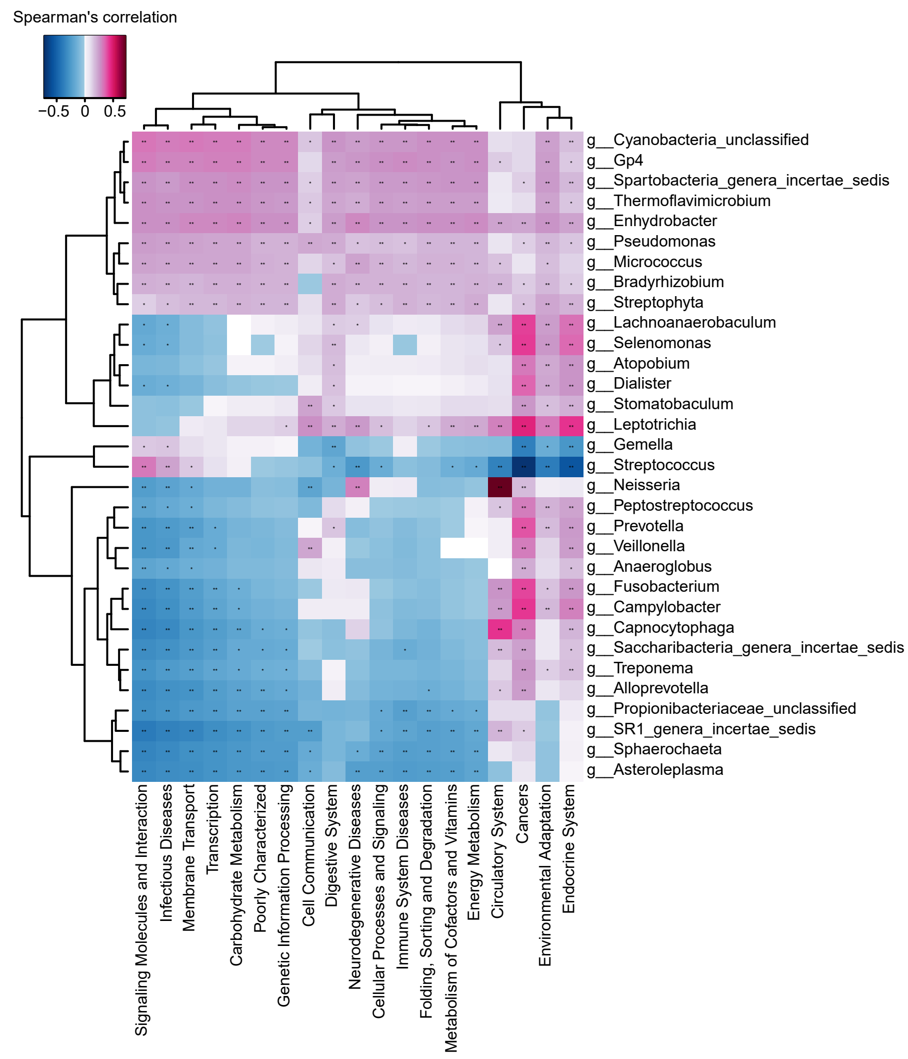
Spearman's correlation
−0.5 0 0.5
g__Cyanobacteria_unclassified
g__Gp4
g__Spartobacteria_genera_incertae_sedis
g__Thermoflavimicrobium
g__Enhydrobacter
g__Pseudomonas
g__Micrococcus
g__Bradyrhizobium
g__Streptophyta
g__Lachnoanaerobaculum
g__Selenomonas
g__Atopobium
g__Dialister
g__Stomatobaculum
g__Leptotrichia
g__Gemella
g__Streptococcus
g__Neisseria
g__Peptostreptococcus
g__Prevotella
g__Veillonella
g__Anaeroglobus
g__Fusobacterium
g__Campylobacter
g__Capnocytophaga
g__Saccharibacteria_genera_incertae_sedis
g__Treponema
g__Alloprevotella
g__Propionibacteriaceae_unclassified
g__SR1_genera_incertae_sedis
g__Sphaerochaeta
g__Asteroleplasma
Signaling Molecules and Interaction
Infectious Diseases
Membrane Transport
Transcription
Carbohydrate Metabolism
Poorly Characterized
Genetic Information Processing
Cell Communication
Digestive System
Neurodegenerative Diseases
Cellular Processes and Signaling
Immune System Diseases
Folding, Sorting and Degradation
Metabolism of Cofactors and Vitamins
Energy Metabolism
Circulatory System
Cancers
Environmental Adaptation
Endocrine System

B

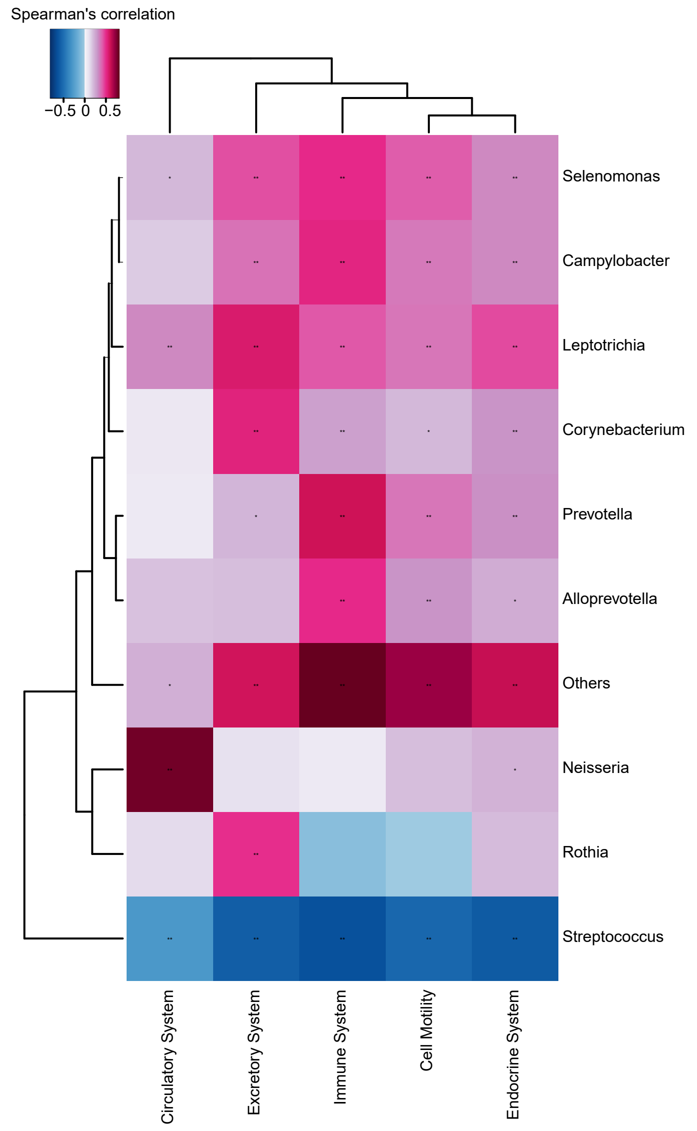
Spearman's correlation
−0.5 0 0.5
Selenomonas
Campylobacter
Leptotrichia
Corynebacterium
Prevotella
Alloprevotella
Others
Neisseria
Rothia
Streptococcus
Circulatory System
Excretory System
Immune System
Cell Motility
Endocrine System

C

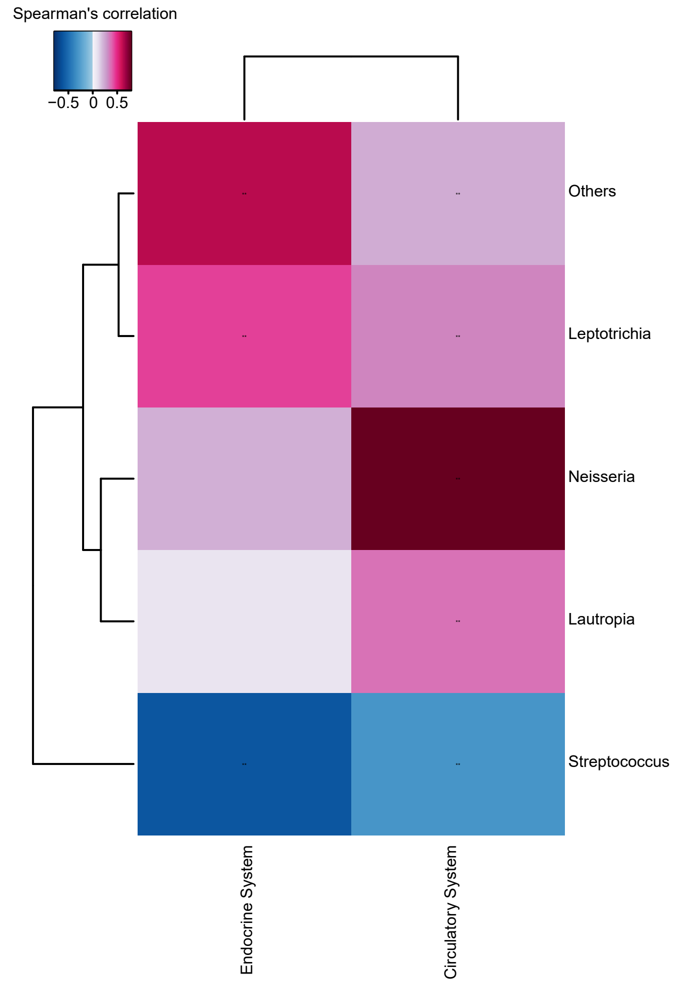
Spearman's correlation
−0.5 0 0.5
Others
Leptotrichia
Neisseria
Lautropia
Streptococcus
Endocrine System
Circulatory System

A

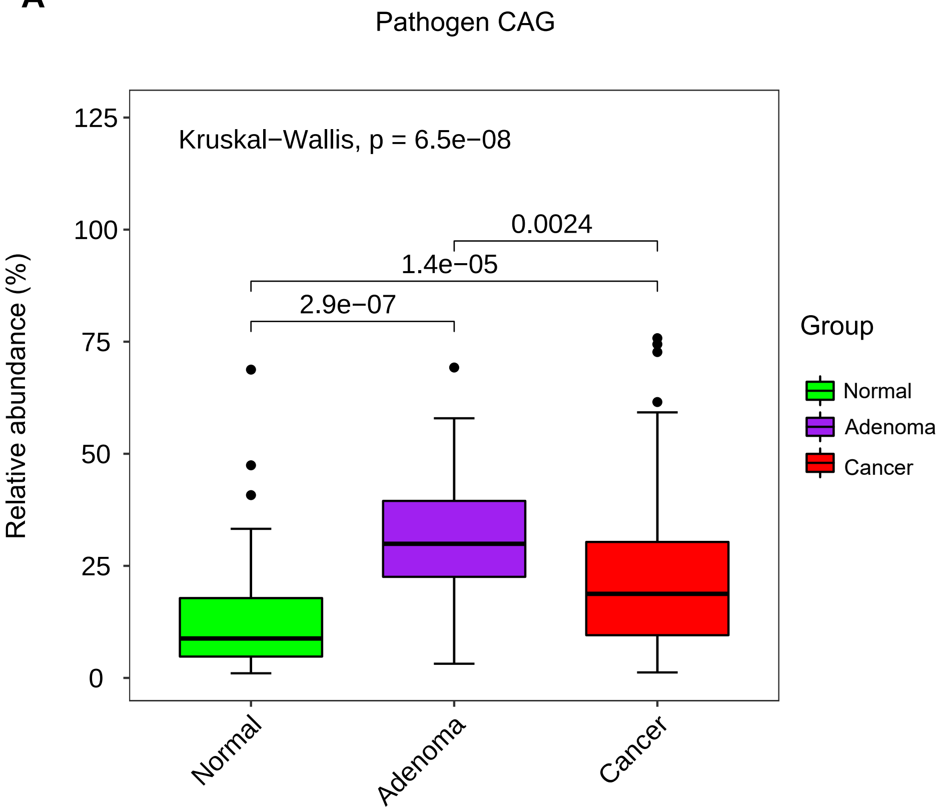
Pathogen CAG
Kruskal−Wallis, p = 6.5e−08
0.0024
1.4e−05
2.9e−07
Relative abundance (%)
125
100
75
50
25
0
Normal
Adenoma
Cancer
Group
Normal
Adenoma
Cancer

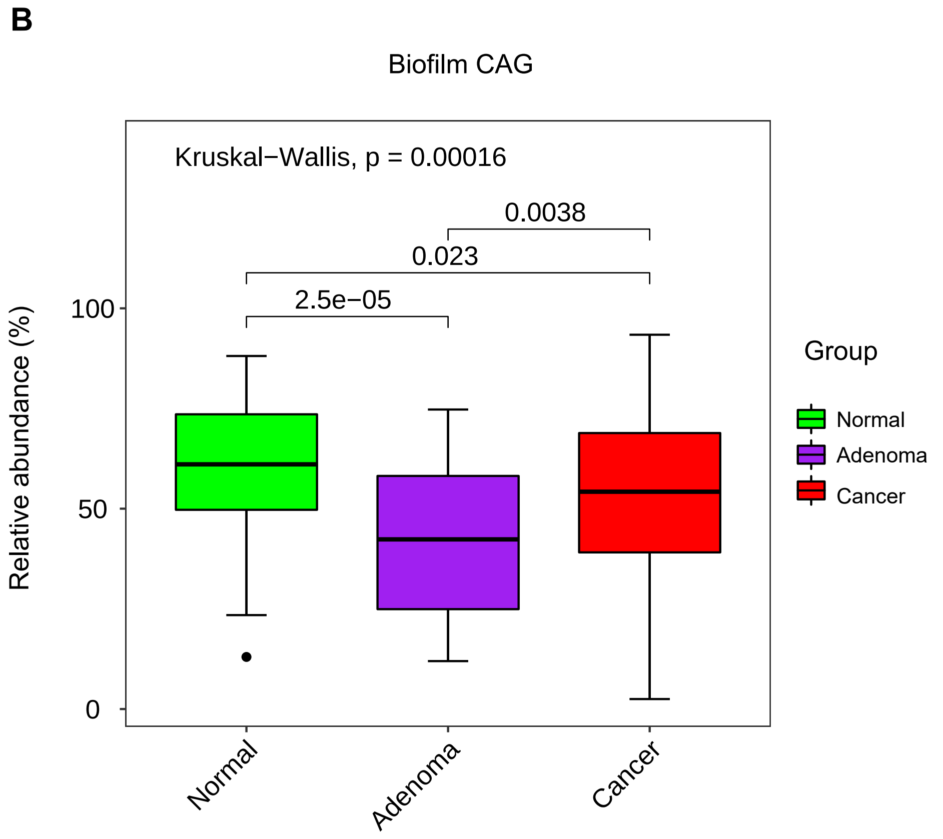
B
Biofilm CAG
Kruskal−Wallis, p = 0.00016
0.0038
0.023
2.5e−05
Relative abundance (%)
100
50
0
Normal
Adenoma
Cancer
Group
Normal
Adenoma
Cancer
