# Supplementary material for: Human oral microbiome dysbiosis as a novel non-invasive biomarker in detection of colorectal cancer
Source: Theranostics. 2020 Sep 18;10(25):11595–606. doi: 10.7150/thno.49515 (PMC7545992; doi:10.7150/thno.49515)
Supplement: Supplementary file 2 — Supplementary tables. [file thnov10p11595s2.zip › s2.docx]

**Table S1.** Relative abundance of 2181 OTUs in all samples. OTUs, operational taxonomic units.

**Table S2.** Oral microbial diversity index in all samples.

**Table S3.** Different bacterial phyla among the CRA cohort, CRC cohort, and healthy controls cohort. CRA, colorectal adenoma; CRC, colorectal cancer.

**Table S4.** Abundance of the different bacterial phyla (log_2_FC, *p* value and q value) between the CRA cohort and CRC cohort. CRA, colorectal adenoma; CRC, colorectal cancer; FC, fold change.

**Table S5.** Abundance of the different bacterial genera (log_2_FC, *p* value and q value) between the CRA cohort and CRC cohort. CRA, colorectal adenoma; CRC, colorectal cancer; FC, fold change.

**Table S6.** Abundance of the different bacterial phyla (log_2_FC, *p* value and q value) between CRA cohort and healthy controls cohort. CRA, colorectal adenoma; FC, fold change.

**Table S7.** Abundance of the different bacterial genera (log_2_FC, *p* value and q value) between the CRA cohort and healthy controls cohort. CRA, colorectal adenoma; FC, fold change.

**Table S8.** Abundance of the different bacterial phyla (log_2_FC, *p* value and q value) between the CRC cohort and healthy controls cohort. CRC, colorectal cancer; FC, fold change.

**Table S9.** Abundance of the different bacterial genera (log_2_FC, *p* value and q value) between the CRC cohort and healthy controls cohort. CRC, colorectal cancer; FC, fold change.

**Table S10.** Five optimal OTU markers selected in the discovery phase between the CRA cohort and healthy controls cohort. CRA, colorectal adenoma; OTU, operational taxonomic unit.

**Table S11.** Five optimal OTU markers selected in the discovery phase between the CRC cohort and healthy controls cohort. CRC, colorectal cancer; OTU, operational taxonomic unit.
